# Arbuscular Mycorrhizal Fungus Enhances Lateral Root Formation in *Poncirus trifoliata* (L.) as Revealed by RNA-Seq Analysis

**DOI:** 10.3389/fpls.2017.02039

**Published:** 2017-11-29

**Authors:** Weili Chen, Juan Li, Honghui Zhu, Pengyang Xu, Jiezhong Chen, Qing Yao

**Affiliations:** ^1^Guangdong Province Key Laboratory of Microbial Signals and Disease Control, Guangdong Engineering Research Center for Litchi, College of Horticulture, South China Agricultural University, Guangzhou, China; ^2^Department of Horticulture, Zhongkai University of Agriculture and Engineering, Guangzhou, China; ^3^State Key Laboratory of Applied Microbiology Southern China, Guangdong Provincial Key Laboratory of Microbial Culture Collection and Application, Guangdong Institute of Microbiology, Guangzhou, China

**Keywords:** arbuscular mycorrhizal fungus, trifoliate orange, lateral root formation, RNA-Seq, P metabolism, sugar metabolism, plant hormone

## Abstract

Arbuscular mycorrhizal fungi (AMF) establish symbiosis with most terrestrial plants, and greatly regulate lateral root (LR) formation. Phosphorus (P), sugar, and plant hormones are proposed being involved in this regulation, however, no global evidence regarding these factors is available so far, especially in woody plants. In this study, we inoculated trifoliate orange seedlings (*Poncirus trifoliata* L. Raf) with an AMF isolate, *Rhizophagus irregularis* BGC JX04B. After 4 months of growth, LR formation was characterized, and sugar contents in roots were determined. RNA-Seq analysis was performed to obtain the transcriptomes of LR root tips from non-mycorrhizal and mycorrhizal seedlings. Quantitative real time PCR (qRT-PCR) of selected genes was also conducted for validation. The results showed that AMF significantly increased LR number, as well as plant biomass and shoot P concentration. The contents of glucose and fructose in primary root, and sucrose content in LR were also increased. A total of 909 differentially expressed genes (DEGs) were identified in response to AMF inoculation, and qRT-PCR validated the transcriptomic data. The numbers of DEGs related to P, sugar, and plant hormones were 31, 32, and 25, respectively. For P metabolism, the most up-regulated DEGs mainly encoded phosphate transporter, and the most down-regulated DEGs encoded acid phosphatase. For sugar metabolism, the most up-regulated DEGs encoded polygalacturonase and chitinase. For plant hormones, the most up-regulated DEGs were related to auxin signaling, and the most down-regulated DEGs were related to ethylene signaling. PLS-SEM analysis indicates that P metabolism was the most important pathway by which AMF regulates LR formation in this study. These data reveal the changes of genome-wide gene expression in responses to AMF inoculation in trifoliate orange and provide a solid basis for the future identification and characterization of key genes involved in LR formation induced by AMF.

## Introduction

Root is essential for plant growth and development, serving a multiple functions including anchorage, absorption of mineral nutrients and water, and production of exudates with growth regulatory properties (Bailey et al., 2002; Chen et al., 2017). The characteristic of root system is mainly presented as root system architecture (RSA), namely the spatial configuration of root system such as root angle, root branching, root diameter and so on (Lynch, 1995). Among all the components of plant RSA, lateral root (LR), formed post-embryonically by LR primordium derived from specific cells of pericycle (Vilches-Barro and Maizel, 2015), is the most important (Nibau et al., 2008; Vilches-Barro and Maizel, 2015; Zhao et al., 2015). RSA is highly plastic, responding to the soil water and nutrient availability, soil matrix heterogeneity, and biotic interactions (Péret et al., 2009; Chen W. L. et al., 2016). As an example for the latter, a mutualistic association develops between roots and arbuscular mycorrhizal fungi (AMF) (Smith and Read, 2008), which comprehensively affect plant RSA, especially the LR formation (Gutjahr and Paszkowski, 2013; Jiang et al., 2015; Chen W. L. et al., 2016). Fusconi (2014) proposed a comprehensive model that AMF affect LR formation probably through three pathways: regulating phosphorus (P) metabolism (increased absorption), regulating carbohydrate metabolism (changed carbohydrate partitioning and pathway), and regulating plant hormone metabolism (modulation of concentration, transport and sensitivity) in host plants. This proposal has been partially verified (Jiang et al., 2015; Chen et al., 2017), however, a comprehensive verification is needed and the contribution of each pathway is also to be quantified.

Plant RSA is strictly controlled by genes, and recently, some experiments revealed the molecular mechanisms underlying the modified RSA with the RNA-Seq technique. Gutjahr et al. (2015) showed that genes expressed in crown root of rice (*Oryza sativa* L.) were related to plant hormone and secondary cell wall metabolism, while they were related to nutrient transportation in large LRs and fine LRs. After AMF inoculation, colonized crown roots adopted an expression profile more related to the mycorrhizal large LRs than to non-colonized crown roots, suggesting a reprogramming of crown root character by AMF. Another transcriptomic analysis of maize (*Zea mays* L.) root showed that, under local nitrate supply, different root types produced different gene expression models, resulting in various patterns of LR formation (Yu et al., 2016). Additionally, gene network was obtained to uncover the mechanism of the adventitious root formation of carnation (*Dianthus caryophyllus* L.) cutting in response to exogenous auxin (Villacorta-Martín et al., 2015). These results demonstrate the potential application of RNA-Seq technique to reveal the comprehensive pathway by which AMF regulates LR formation.

So far, most studies with RNA-Seq technique focused on herbaceous plant roots (Nawy et al., 2005; Brady et al., 2007; Zhang et al., 2014; Gao et al., 2015; Opitz et al., 2016; Song et al., 2016), and in contrast, few study shed light on the woody plants. Citrus, one of the most important woody fruit crops worldwide, strongly depends on AMF for acquiring nutrients because of lacking of root hairs (Wu et al., 2016). Previous studies indicated the significant regulation of LR formation in citrus plants by AMF (Yao et al., 2009; Chen et al., 2017). In this study, we chose trifoliate orange (*P. trifoliata* L. Raf), commonly used as the rootstock in the citrus-producing areas of China (Gao et al., 2016), as host plants to establish symbiosis with AMF. We aimed to dissect the pathways by which AMF regulates LR formation at molecular level with RNA-Seq technique, and to probe into the respective contribution of different pathway to the regulation.

## Materials and methods

### Experimental material and experimental design

To establish AM symbiont in pot culture, the commercially obtained trifoliate orange (*P. trifoliata* L. Raf) seeds were used as host plants, and AMF isolate was *Rhizophagus irregularis* BGC JX04B provided by Beijing Academy of Agriculture and Forestry Sciences (Chen et al., 2017). *R. irregularis* inocula were propagated with clover (*Trifolium repense* L.) and sorghum (*Sorghum bicolor*) as hosts for 4 months in the greenhouse. At harvest, the spore density was quantified to be ca. 49 spores per gram inoculum. The growth substrate was the mixture of autoclaved (121°C, 2 h) soils and peat (1:2, v:v). The soils were collected from the experimental orchard of South China Agricultural University, and the soil chemical properties were determined as follows: pH 4.60, organic matter content 1.58%, available N 65.0 mg·kg^−1^, available P 20.5 mg·kg^−1^, and available K 57.1 mg·kg^−1^. The substrate was additionally applied with 200 mg·kg^−1^ N (NH_4_NO_3_), 10 mg·kg^−1^ P (KH_2_PO_4_), and 100 mg·kg^−1^ K (KNO_3_).

We conducted two pot experiments to reveal the influence of AMF inoculation on LR formation of trifoliate seedlings. The first experiment was set up for RNA-Seq analysis with two treatments [non-mycorrhizal (C) and mycorrhizal (T)]. Each treatment comprised of 3 replicates. Each pot was filled with 450 g substrate and 50 g AMF inocula (T treatment) or sterilized inocula (C treatment). The second experiment was set up with the same treatments as in the first experiment, and the sugar contents in roots as affected by AMF inoculation were investigated.

Trifoliate orange seeds were surface-sterilized with 70% ethanol for 15 min, rinsed in distilled water for 5 times, and then germinated in autoclave-sterilized peat at 28°C in dark. Seedlings with five leaves and similar vigor were selected and transplanted to pots (two seedlings in each pot). All mycorrhizal and non-mycorrhizal seedlings grew in the greenhouse under natural light conditions with 22~30°C and 60%~80% relative humidity. Plants were harvested at 4 months after transplanting.

### Harvest and physio-biochemical parameters measurement

At harvest, shoots and roots were separated and the fresh weights were recorded respectively, then the LR numbers of different orders were counted and LR forming capacity (LRC) was calculated as the value of (the number of *N* order LR)/(the number of *N-1* order LR). LR tips (about 1 cm length) were randomly selected and cut, and preserved (about 0.2 g) at −80°C for RNA-Seq and qRT-PCR analysis. The leftover roots were cut into fragments (about 1 cm length) and 50 fragments were randomly picked up for the measurement of mycorrhizal colonization. Other root fragments were used for carbohydrate determination.

The chlorophyll contents in leaves were measured according to Chen Z. et al. (2016). After digested with HNO_3_, the shoot P contents were measured by a spectrophotometer according to Arshad et al. (2016). Mycorrhizal staining was performed according to Phillips and Hayman (1970) and mycorrhizal colonization was quantified according to Biermann and Linderman (1981). The carbohydrate contents (sucrose, glucose, and fructose) in primary roots and LR were colorimetrically determined according to Wu et al. (2015) with some modification. Briefly, 100 mg fresh roots were homogenized with 4 mL 80% ethanol, incubated for 30 min at 80°C, and then centrifuged at 8,000 rpm for 10 min. The residues were extracted twice as described above, and all the supernatants were combined for the analysis of carbohydrate content. Sucrose was assayed with a mixture of 0.4 mL supernatant and 0.2 mL 2 mol·L^−1^ NaOH at 100°C for 5 min, which were then mixed together with 2.8 mL 5 mol·L^−1^ HCl and 0.8 mL 0.1% resorcinol at 100°C for 10 min followed by measurement of absorbance at 480 nm. Glucose concentration was determined by mixing 1 mL of supernatant with 2 mL of prepared solution (1 mg·mL^−1^ o-dianisidine dihydrochloride, 1 mg·mL^−1^ horseradish peroxidase, and 1 U·mL^−1^ glucose oxidase) at 30°C for 5 min, then added 4 mL 10 mol·L^−1^ H_2_SO_4_ solution to terminate the reaction, and followed by measurement of absorbance at 460 nm. Fructose was assayed with a mixture of 0.4 mL supernatant, 0.8 mL 0.1% resorcinol and 0.4 mL 5 mol·L^−1^ HCl at 100°C for 10 min followed by measurement of absorbance at 480 nm. The carbohydrate contents were quantified according to the linear standard curves constructed with respective sugars.

### RNA-seq and transcriptomic analysis

Total RNA in the root tips from C and T treatments (3 independent replicates in each treatment) was isolated with RNA extraction kit (Huayueyang Biotechnology Co., LTD, Beijing) according to the manufacturer's protocol. The RNA integrity was checked with Agilent 2100 Bioanalyzer and Agilent RNA 6000 Nano Kit, and RNA concentration was determined using a Nanodrop 2000 spectrophotometer (NanoDrop Technologies, Wilmington, DE, USA). One aliquot was sent to Annoroad Gene Technology Co. Ltd. (Beijing, China) for RNA-seq. The libraries were sequenced on an Illumina Hiseq™ 4000 platform and 150 bp paired-end reads were generated.

After removing reads containing adapters or more than 50% N and low-quality reads from the raw reads, clean reads were obtained for all subsequent analyses. The *Citrus sinensis* reference genome (Xu et al., 2012), directly downloaded from the citrus genome website (http://citrus.hzau.edu.cn/orange/), was used for the mapping of clean reads by TopHat (Trapnell et al., 2009).

The RPKM value (Reads Per Kilobase per Million reads) and Fold Change value for each gene were measured by applying GFOLD algorithm (Feng et al., 2012). Differential expression analysis of two treatments was performed using the DESeq (Anders, 2010). The threshold for significantly differential expression was set as follows: adjusted *P* < 0.05 and |log2(fold change)| > 1 (Anders and Huber, 2012). The *P*-values were adjusted using the Benjamini and Hochberg method (Benjamini and Hochberg, 1995). The volcano plot of differentially expressed genes (DEGs) and cluster diagrams were made by R packages “gplots” and “ggplot2,” respectively.

Gene ontology (GO) enrichment analysis of DEGs was implemented by the Blast2GO software (Conesa et al., 2005). Based on Kyoto encyclopedia of genes and genomes (KEGG, http://www.genome.jp/kegg/), the KOBAS 2.0 software (http://kobas.cbi.pku.edu.cn/) was used to test the statistical enrichment of DEG in KEGG pathways (Xie et al., 2011).

### Quantitative real-time PCR

Another aliquot of extracted RNA was used for the quantitative real-time PCR (qRT-PCR) according to previous protocol (Chen et al., 2017), with 3 independent replicates for each treatment. Briefly, cDNA was synthesized with the extracted RNA as template using iScript™ cDNA Synthesis Kit (Bio-Rad, USA). *18SrRNA* gene was used as internal reference (Yan et al., 2012) in this study. All gene-specific primers designed with Batchprimer3 (https://probes.pw.usda.gov/batchprimer3/) were described in Supplementary Table 1 and the specificity was checked using Blasting Tool of NCBI. Primers were synthesized by Shanghai Sangon BioTech Co. PCR was conducted with iTaq™ Universal SYBR Green Supermix Kit (Bio-Rad, USA) using CFX96 Real-Time System. Each reaction mixture was 20 μL containing 5 μL diluted cDNA template, 250 nmol·L^−1^ of forward and backward primers, 10 μl SYBR Green supermix and 4 μL sterilized ddH_2_O. The two-step qRT-PCR was run as follows: 95°C for 30 s, 39 cycles of 95°C for 5 s, and 60°C for 30 s in 96-well plates (Bio-rad, USA). All samples were amplified in triplicate from the same RNA preparation and the mean value was considered. The relative expression of each target gene was calculated by using 2^−ΔΔCt^ method (Livak and Schmittgen, 2001).

### Statistical analysis

All data were presented as mean ± standard error of three replicates. The *t*-test was performed and the correlations between the LR numbers and DEGs were calculated with bivariate correlations analysis using IBM SPSS v.21 statistical software (SPSS Inc., Chicago, IL).

To identify *PT* (encoding phosphate transporter) genes induced by AMF, phylogeny tree was constructed with the DEG data against AMF-induced *PT* genes in citrus reported previously (Shu et al., 2012) by using MEGA6 software. To discover the correlations between DEGs and LR formation, redundancy analysis (RDA) was performed using CANOCO 4.5 statistical package (Braak and Smilauer, 2002), and the results were visualized with the extension CanoDraw. To explore the relationships between AMF inoculation, LR formation, and metabolisms of P, sugar and plant hormones, a hypothetical model was specified and analyzed with Partial Least Squares Structural Equation Modeling (PLS-SEM) with the support of the SmartPLS 2.0 M3 software (Ringle et al., 2005). The standardized path coefficient values were generated with the PLS algorithm using Path weighting scheme, and a bootstrapping method was used to obtain the significance of path coefficients.

## Results

### Mycorrhizal colonization and plant growth

In the inoculated roots (T treatment), the mycorrhizal colonization was 28.99% (Table 1), while no mycorrhizal structures was observed in the non-inoculated roots (C treatment).

**Table 1:** Influence of AMF on the growth of trifoliate orange seedlings.

**Treatment**	**Shoot FW (g)**	**Root FW (g)**	**R/S**	**Mycorrhizal colonization (%)**
C	0.38 ± 0.04	0.79 ± 0.11	2.11 ± 0.10	0.00 ± 0.00
T	1.01 ± 0.07^**^	1.15 ± 0.12^**^	1.17 ± 0.11^**^	28.99 ± 0.89^***^

C, non-mycorrhizal treatment; T, mycorrhizal treatment; FW, fresh weight; R/S, root biomass/shoot biomass; For each column, values (mean ± standard error of three replicates) followed by

“**” and “***” *mean significant difference at P < 0.01 and P < 0.001, respectively*.

**Figure 1:**
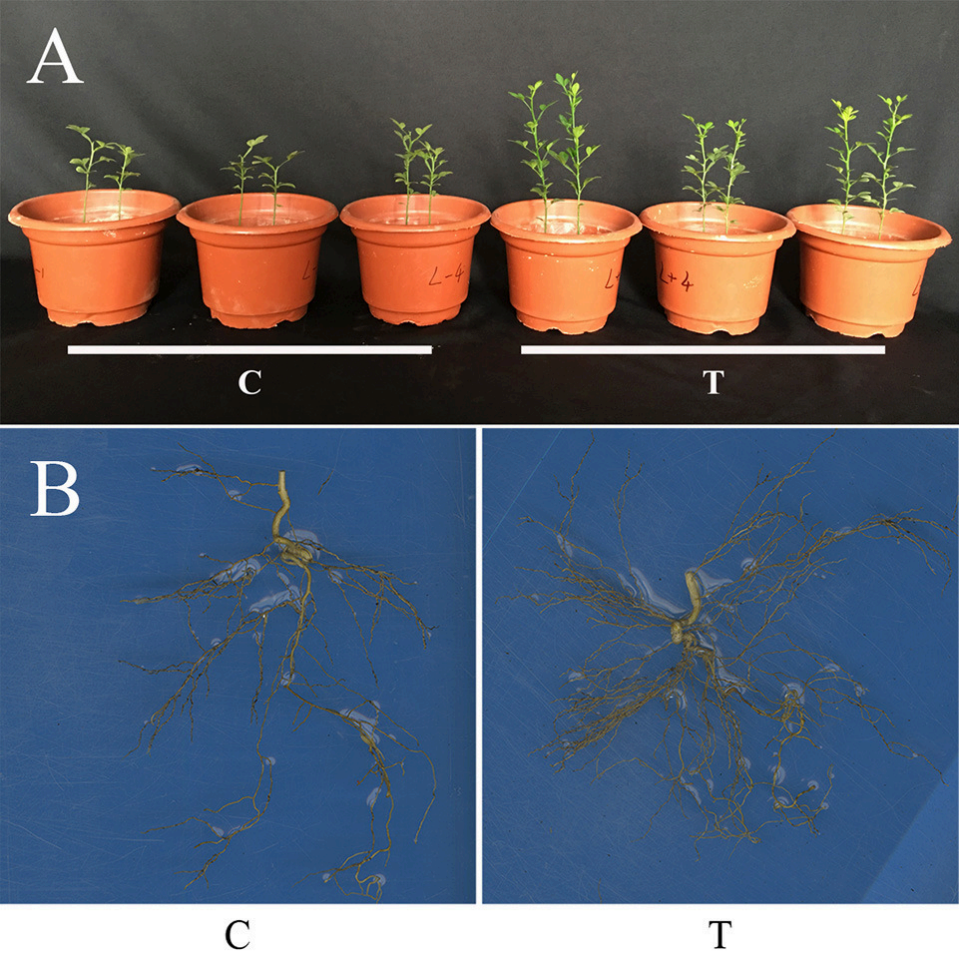
Plant growth of trifoliate orange seedlings as affected by AMF inoculation. **(A)** Photo showing the overall plant growth; **(B)** The root systems. C: non-mycorrhizal treatment, T: mycorrhizal treatment.

Compared with the non-mycorrhizal treatment (C), AMF inoculation significantly increased the growth of trifoliate orange seedlings, as revealed by the increase in either shoot FW or root FW, but decreased the R/S (Table 1, Figure 1A). It was clear that the root system were more vigorous and complex after AMF inoculation (Figure 1B).

**Figure 2:**
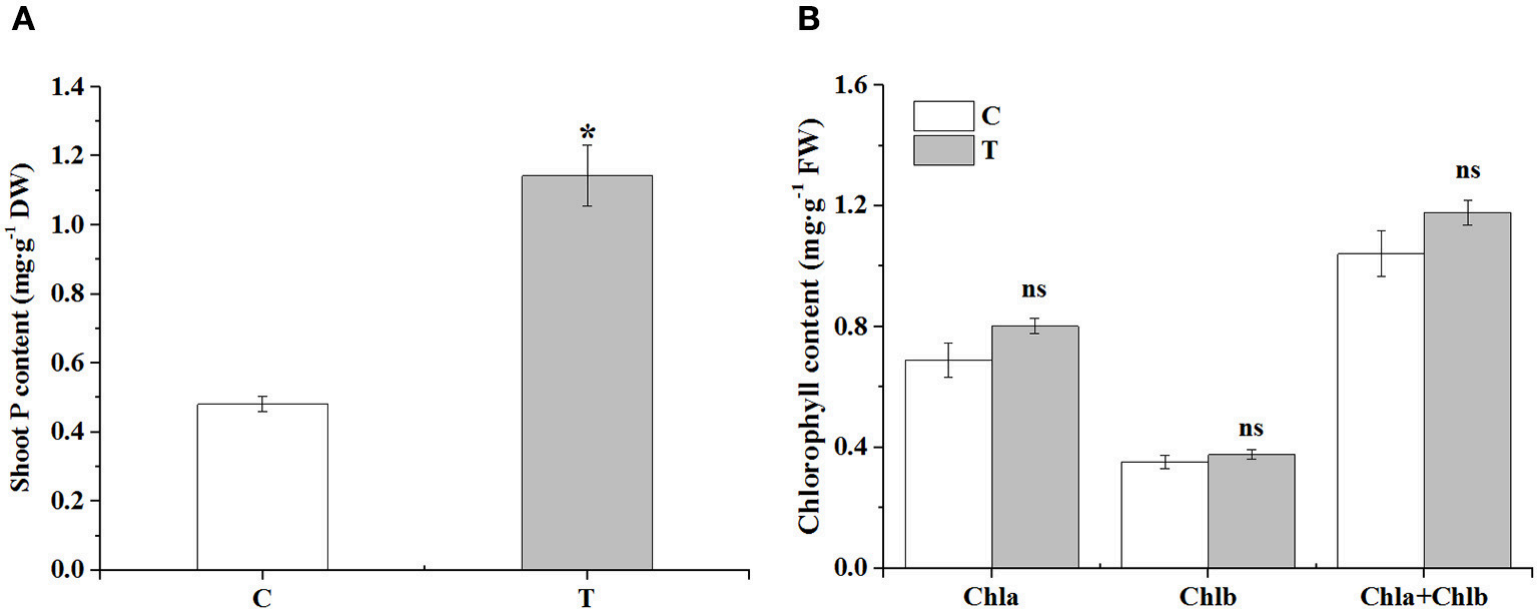
The effects of AMF on shoot P contents and leaf chlorophyll contents. **(A)** Shoot P contents; **(B)** Leaf chlorophyll contents. C: non-mycorrhizal treatment, T: mycorrhizal treatment; DW: dry weight, FW: fresh weight; “*” and “ns” mean significant difference at *P* < 0.05 and no significant difference, respectively.

In the aboveground part, the phosphorus content was significantly increased in the mycorrhizal plants, over two times higher than that in the non-mycorrhizal plants (Figure 2A). There was no obvious difference in the contents of chlorophyll a, b and total chlorophyll between the two treatments, although AMF inoculation slightly increased these parameters (Figure 2B).

### Enhancement of lateral root formation by AMF inoculation

AMF inoculation greatly increased the LR formation except for the 1st order LR (Table 2). In detail, the 2nd order LR number was increased by nearly one time, and the 3rd order LR number was increased by over 3 times. The 4th order LR number was about 5 for the mycorrhizal plants, while no 4th order LR occurred for the non-mycorrhizal plants (Table 2). Moreover, AMF inoculation significantly elevated the formation capacity of the 2nd and 3rd LR (Table 2).

**Table 2:** Influence of AMF on lateral root formation in trifoliate orange seedlings.

**Treatment**	**Lateral root number of different orders**					**LR formation capacity**		
	**1st LR**	**2nd LR**	**3rd LR**	**4th LR**	**Total LR**	**1st LR**	**2nd LR**	**3rd LR**
C	25.00 ± 2.08	63.83 ± 2.05	9.67 ± 2.68	0.00 ± 0.00	98.50 ± 6.50	2.58 ± 0.14	0.15 ± 0.04	0.00 ± 0.00
T	29.33 ± 3.24^ns^	101.33 ± 4.28^***^	37.17 ± 1.74^**^	4.83 ± 1.20^*^	172.67 ± 7.31^**^	3.53 ± 0.36^ns^	0.37 ± 0.01^**^	0.13 ± 0.03^**^

C, non-mycorrhizal treatment; T, mycorrhizal treatment; LR, lateral root; For each column, values (mean ± standard error of three replicates) followed by

“*”, “**”, “***”and “ns” *mean significant difference at P < 0.05, P < 0.01, P < 0.001 and no significant difference, respectively*.

To verify the effect of AMF inoculation on the LR formation, we conducted a separate experiment, and got the similar results, where AMF inoculation also significantly increased numbers of the 1st order LR and the 3rd order LR (Supplementary Figure 1A). Meanwhile, the contents of glucose and fructose in the primary root were significantly increased (Supplementary Figure 1B) and the sucrose content in lateral roots was significantly increased by AMF inoculation (Supplementary Figure 1C).

### Bioinformatics analysis of RNA-seq data and identification of DEGs in roots

**Figure 3:**
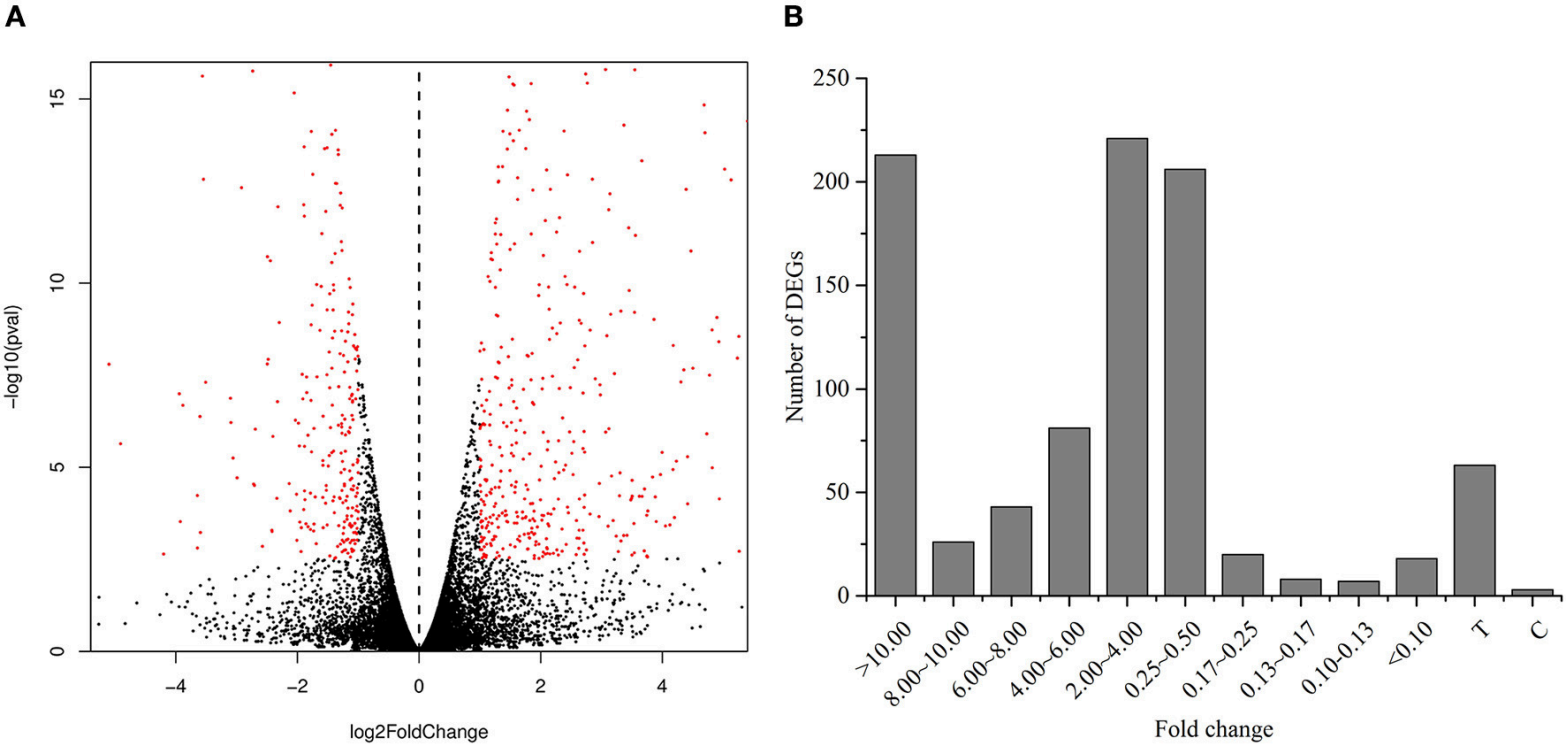
Analysis of differentially expressed genes between the mycorrihzal and non-mycorrhizal treatment. **(A)** Volcano plot showing differentially expressed genes. Red dots in left and right indicate the significantly down-regulated and up-regulated genes, respectively. **(B)** Number of DEGs grouped by range of fold change. C: non-mycorrhizal treatment, T: mycorrhizal treatment; “C” and “T” mean these genes only found to be expressed in C and T treatment, respectively.

We performed RNA-Seq analysis to reveal the molecular mechanisms involved in the regulation of LR formation by AMF inoculation. An overview of the sequencing and assembly was outlined in Table 3. High-quality reads (clean reads) were obtained after trimming and applying quality filter, ranging from 40,163,170 to 41,436,784 reads for individually sequenced libraries. On the whole, about 80% of these reads (Table 3) mapped into the genome of *Citrus sinensis* were used to the subsequent analysis. The RNA-Seq dataset was deposited in NCBI Short Read Archive (SRA; http://www.ncbi.nlm.nih.gov/sra) under the accession number SRP119883. The published citrus genome includes 28,195 genes, and in present work, we detected the expressions of 16,383 genes in trifoliate orange root tips, including all experimental replicates. By comparing their expression levels under mycorrhizal and non-mycorrhizal treatment, 909 DEGs were identified (see in Supplementary Data 1), among which 647 were up regulated, and 262 down regulated by AMF inoculation (Figure 3A). Grouping by fold change, most DEGs distributed in the range of >10 (23.43%), 2~4 (24.31%), and 0.25~0.50 (22.66%) fold. 64.25% of DEGs were up regulated by AMF inoculation, not including the uniquely expressed genes (6.93%) in the mycorrhizal root tips (Figure 3B), while less than one third of DEGs (28.49%, Figure 3B) was down regulated in response to AMF inoculation. Additionally, only 3 genes were specifically expressed in the non-mycorrhizal plants, in contrast to 63 genes specifically expressed in mycorrhizal plants. Among all DEGs, 121 genes were uncharacterized according to the reference genome (Supplementary Data 1). The most 10 up-regulated and down-regulated genes were listed in Table 4. It is worthy to note that gene (*LOC102613232*) encoding inorganic phosphate transporter was up-regulated by almost 2000 times by AMF.

**Table 3:** Results of RNA sequencing and mapping.

**Sample ID**	**Raw reads**	**Clean reads**	**Clean reads rate (%)**	**Raw Q30 bases rate (%)**	**Clean Q30 bases rate (%)**	**Mapped reads**	**Mapped unique reads**	**Mapping ratio (%)**
C1	40,717,624	40,227,124	98.80	94.55	94.83	32,261,488	28,872,865	80.20
C2	42,065,554	41,436,784	98.50	94.19	94.55	33,014,855	29,735,224	79.68
C3	41,360,176	40,577,880	98.11	93.18	93.72	32,278,591	29,203,585	79.55
T1	40,741,198	40,163,170	98.58	94.65	94.93	32,547,365	29,715,831	81.04
T2	41,581,390	40,986,896	98.57	94.25	94.57	33,212,525	30,289,274	81.03
T3	41,521,274	40,935,806	98.59	94.26	94.58	33,294,465	30,251,475	81.33

*C, non-mycorrhizal treatment; T, mycorrhizal treatment*.

**Table 4:** The most 10 up-regulated and down-regulated genes in DEGs.

**Up-regulated genes**			**Down-regulated genes**		
**Gene ID**	**Annotation**	**Fold Change**	**Gene ID**	**Annotation**	**Fold change**
LOC107177624	putative bark agglutinin LECRPA3	4538.985	LOC107178258	uncharacterized LOC107178258	0.071
LOC102614727	ATPase 11, plasma membrane-type-like	3649.422	LOC102606964	aspartic proteinase nepenthesin-1-like	0.069
LOC102616732	senescence-specific cysteine protease SAG39	3070.634	LOC102623053	BEL1-like homeodomain protein 11	0.068
LOC102630908	senescence-specific cysteine protease SAG39-like	2280.439	LOC102622143	zinc finger BED domain-containing protein DAYSLEEPER-like	0.068
LOC102616938	senescence-specific cysteine protease SAG39-like	2165.832	LOC107176063	disease resistance protein RPM1-like	0.057
LOC102613232	inorganic phosphate transporter 1-11	2002.312	LOC102627718	uncharacterized LOC102627718	0.035
LOC102616361	putative Serine carboxypeptidase-like 23	1708.107	LOC102626253	cationic peroxidase 1-like	0.030
LOC102612840	methanol O-anthraniloyltransferase-like	1633.213	LOC107176317	zinc finger BED domain-containing protein RICESLEEPER 1-like	0.024
LOC102608280	triacylglycerol lipase 2-like	1617.213	LOC102619921	uncharacterized LOC102619921	0.009
LOC102614186	subtilisin-like protease SBT1.2	1599.513	LOC107175515	uncharacterized LOC107175515	0.005

“*Fold Change” indicates the ratio of expressions in T and C treatment of the same gene*.

### Validation of sequencing results by qRT-PCR

To validate the RNA-Seq data, qRT-PCR of 14 randomly selected genes was performed. The expressions of these genes identified by qRT-PCR were as the same trend as observed in RNA-Seq (Supplementary Figure 2), and there was a significantly positive correlation (*R*^2^ = 0.7661, Supplementary Figure 3) between RPKM values and qRT-PCR data, indicating that the qRT-PCR results of these genes were consistent with their transcript abundance changes detected by RNA-Seq, which means the RNA-Seq data were credible for exploring the mechanism of phenotypic changes of root system induced by AMF inoculation.

### Functional classification of DEGs

All DEGs were assigned to GO categories, involved in 242 biological processes, 102 molecular functions, and 30 cellular components. Top 10 of each category were showed in Supplementary Figure 4. In detail, in the biological process category, oxidation-reduction process (72 DEGs), biological process (35 DEGs) and defense response to fungus (27 DEGs) were strongly represented. In the molecular functional category, 22, 21, and 12 DEGs were identified as belonging to kinase activity, metal ion binding, and oxygen binding subcategories, respectively. In the cellular component category, subcaterories of membrane (59 DEGs) and cytoplasmic membrane-bounded vesicle (46 DEGs) contained more differentially expressed genes.

Pathway enrichment analysis using KOBAS was implemented to analyze the biological functions of DEGs induced by AMF inoculation. Metabolic pathways (96 DEGs) and biosynthesis of secondary metabolites (70 DEGs) were the two enriched pathways comprising the most DEGs (Supplementary Figure 5). In addition, 14 and 7 DEGs were annotated with the starch and sucrose metabolism and plant hormone signal transduction pathways, respectively.

### DEGs related to metabolisms of P, sugar, and plant hormones

**Figure 4:**
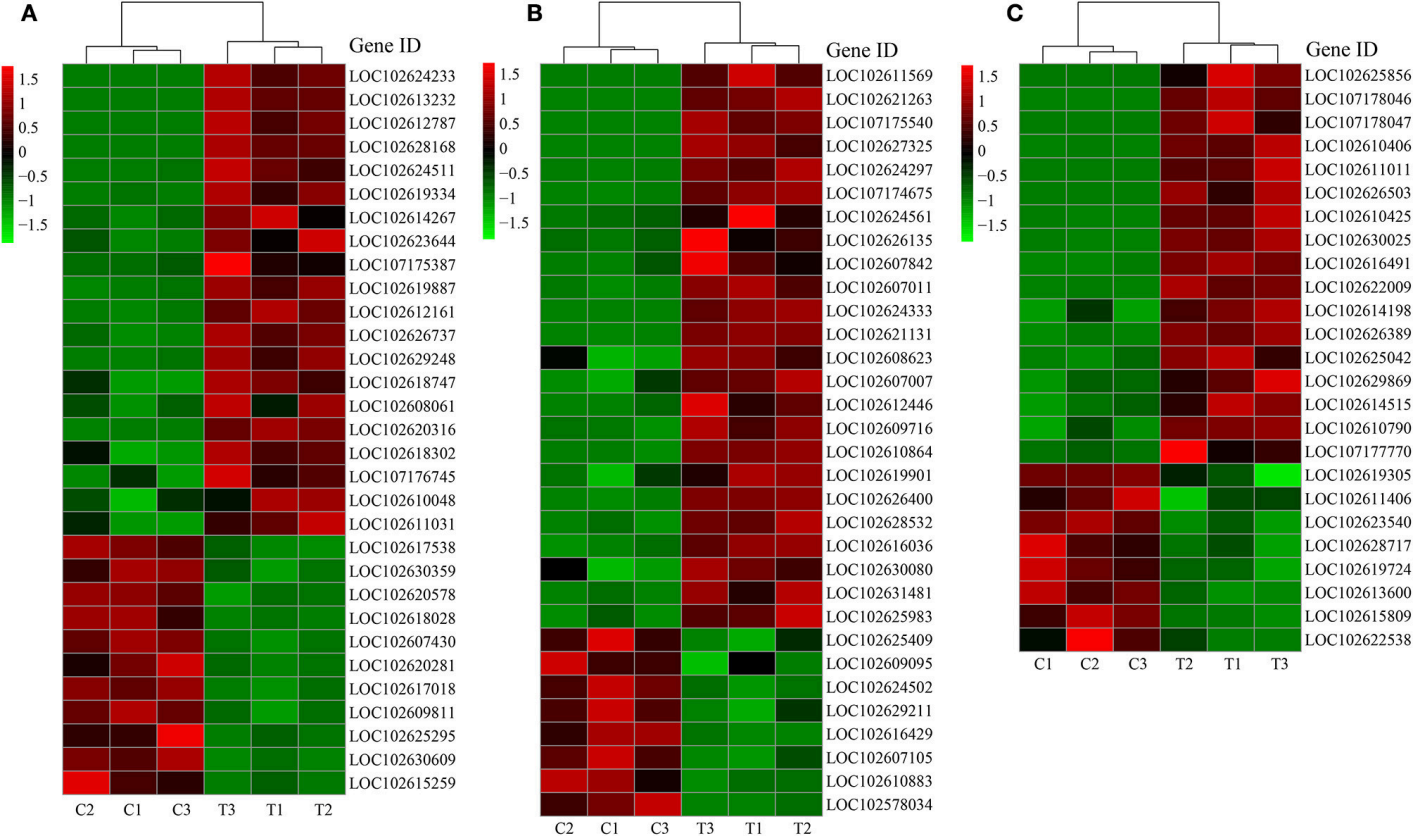
DEGs related to different categories. **(A)** DEGs related to P metabolism; **(B)** DEGs related to sugar metabolism; **(C)** DEGs related to plant hormone. C: non-mycorrhizal treatment, T: mycorrhizal treatment. The annotation of DEGs is listed in Supplementary Data 3.

Due to the close relationship between LR formation and P status, sugar supply, and plant hormone, we selected the genes related to P metabolism, sugar metabolism, and plant hormone for further analysis (Figures 4A–C).

About two-third of the P metabolism related DEGs were up-regulated by AMF inoculation (Figure 4A). Five *PT* genes (*LOC102613232, LOC102628168, LOC102626737, LOC102608061, LOC102609811*) were found as DEGs (DEGs related to phosphorus metabolism). Particularly, the expression of *LOC102613232* and *LOC102628168* in T treatment were over 1000 times more than those in C treatment (Figure 4A). Phylogeny tree indicates that both were AM-induced *PT* genes (Supplementary Figure 6). In contrast, most of the down-regulated genes encoded for acid phosphatase. The expression of 24 out of 32 DEGs related to sugar metabolism were higher in T treatment than in C treatment (Figure 4B). Moreover, 6 genes functioned with phosphorus involved. Interestingly, the most promoted DEGs were associated with polygalacturonase (PG), chitinase (CHI) and their analogs (PG-like protein, CHI-like protein). Three chitinase-related DEGs belong to endochitinase, among which *LOC102607011* was a chitinase class III gene with over 9 times expression level in mycorrhizal root than that in the non-mycorrhizal root. For the genes involved in plant hormone metabolism, 17 were up-regulated and 8 were down-regulated after AMF inoculation (Figure 4C). In details, 10 DEGs were related to auxin, 7 DEGs to ethylene, 4 DEGs to gibberellic acid (GA), 2 DEGs to abscisic acid (ABA), and 2 DEGs to cytokinin, respectively. There were 8, 4, 3, 1, and 1 DEGs related to auxin, ethylene, GA, ABA, and cytokinin were up-regulated by AMF inoculation.

**Figure 5:**
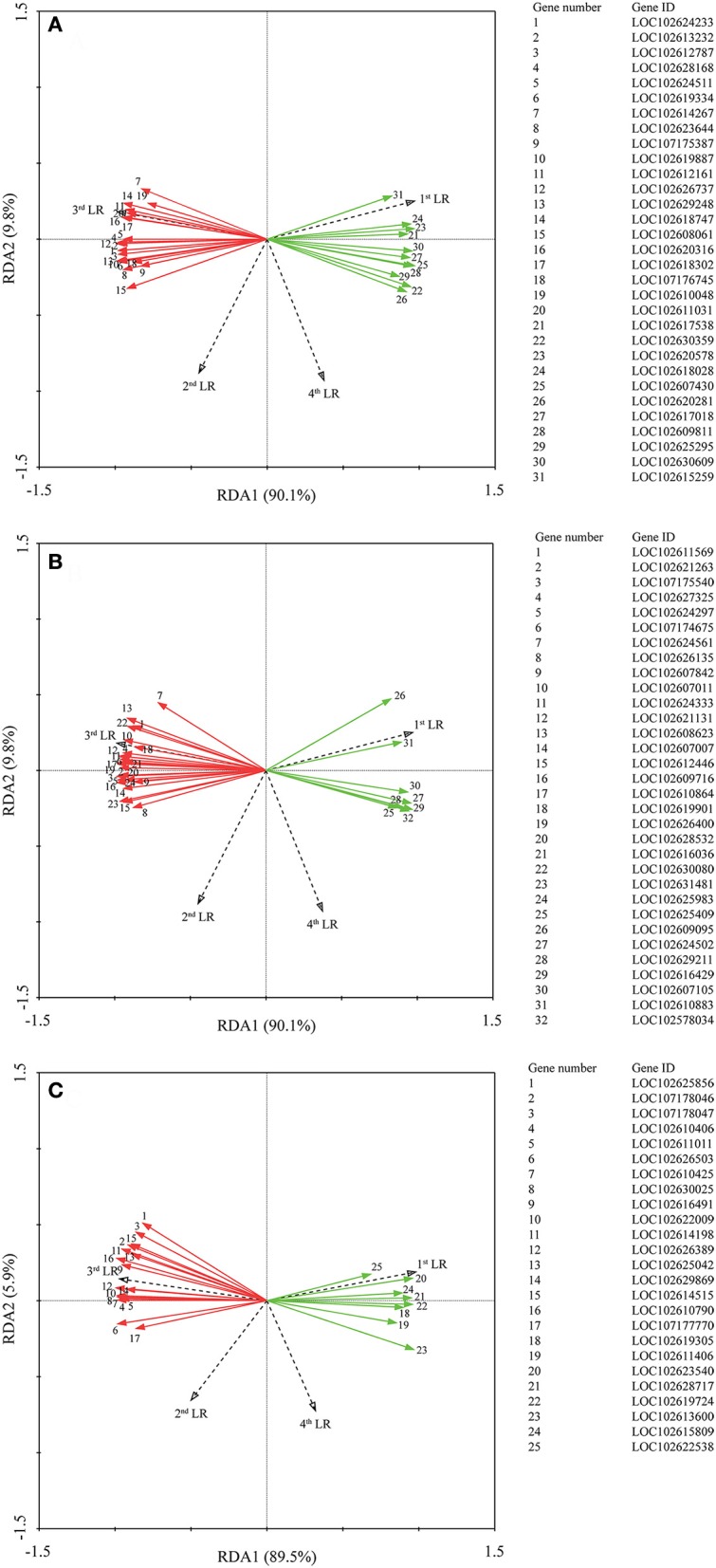
Redundancy analysis (RDA) indicating the correlations between DEGs related to different categories and LR numbers. **(A)** DEGs related to P metabolism. **(B)** DEGs related to sugar metabolism; **(C)** DEGs related to plant hormone. Red and green arrows indicated the up-regulated and down-regulated DEGs due to AMF inoculation, respectively. The annotation of DEGs is listed in Supplementary Data 3.

Correlation analysis (Supplementary Tables 2–4) showed that all genes were not significant correlated with the 1st LR number. Moreover, we performed RDA to investigate the correlation between the LR formation (LR number) and the main factors (P metabolism, sugar metabolism, and plant hormone metabolism). It is interesting that all the down-regulated genes were positively correlated with the 1st LR number, which was not affected by AMF inoculation; and in contrast, all the up-regulated genes were positively correlated with the 3rd LR number, which was significantly promoted by AMF inoculation (Figure 5). In details, for phosphorus metabolism, some genes encoding phosphatase (*LOC102612161, LOC 102618747, LOC102620316, LOC102618302, LOC102611031*) were closely correlated with 3rd LR number (Figure 5A, Supplementary Table 2); for carbohydrate metabolism, some genes encoding chitinase (*LOC102627325, LOC102607011, LOC102621131*) and glucose-6-phosphate 1-dehydrogenase (*LOC102624333*) were closely correlated with 3rd LR number (Figure 5B, Supplementary Table 3); and for plant hormones, some genes (*LOC102622009, LOC102629869, LOC102610790*) involved in auxin metabolism were closely correlated with 3rd LR number (Figure 5C, Supplementary Table 4).

### DEGs related to metabolisms of lipid biosynthesis and metabolism

Totally, 28 DEGs were related to lipid biosynthesis and metabolism, with 11 down-regulated and 17 up-regulated (Supplementary Table 5). Among these DEGs, 7 DEGs were involved in fatty acid metabolism, with 4 up-regulated by AMF, and 8 DEGs were related to steroid biosynthesis, with all strongly up-regulated by AMF. The up-regulated and fatty acid metabolism related genes participated in lipid synthesis (*LOC102626302, LOC102618665, LOC102612001*) and the desaturation reactions (*LOC102611163*).

### Probing into pathways by which AMF inoculation affects LR formation

**Figure 6:**
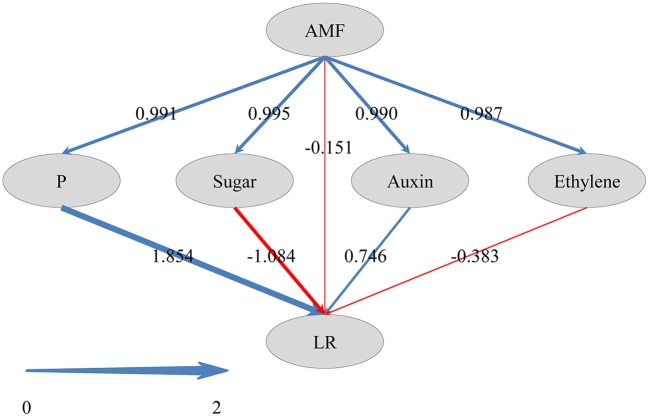
Directed graph of the Partial Least Squares Structural Equation Modeling (PLS-SEM). Larger path coefficients were reflected in the width of the arrows; and blue and red arrows indicate positive and negative effects, respectively.

To reveal how AMF inoculation affected the LR formation via the metabolisms of phosphorus, carbohydrate, and plant hormones, and to gain a comprehensive view of these pathways, we performed PLS-SEM analysis. In PLS-SEM, all latent variables were significant (Supplementary Table 6) and goodness-of-fit measures, such as average variance extracted (AVE; indicator for converge validity) and composite reliability (indicator for internal consistency reliability) were higher than 0.5 and 0.7, respectively (Hair et al., 2011; Supplementary Table 7), indicating this model was reliable. As showed in Figure 6, AMF inoculation promoted the metabolisms of P (0.991), sugar (0.995), auxin (0.990), and ethylene (0.987). The total effect of AMF on LR formation (0.999) was significantly positive (Supplementary Table 6), in which the indirect effects via P metabolism (1.854) and auxin (0.746) were dominant (Figure 6). Besides, the indirect effects via sugar metabolism (−1.084) and ethylene metabolism (−0.383) were negative, and the effect of other unknown pathway (−0.151) was negative.

## Disscussion

In this study, the mycorrhizal colonization of trifoliate orange seedlings colonized by *R. irregularis* was 28.99%, similar to that colonized by *Acaulospora scrobiculata* (15.8%) and *Funneliformis mosseae* (38.9%) in the same plant species (Wu et al., 2016). DEGs related to Ser/Thr receptor kinases (51 DEGs), chitinases (11), lectins (5), and glutathione S-transferases (4) were observed (Supplementary Data 1), which are mycorrhiza-induced genes according to Guether et al. (2009), highlighting the evolutionary conservation of genetic processes supporting such symbiosis.

Promotion of LR formation by AMF has been demonstrated both in annual plants (Ronco et al., 2008; Gutjahr et al., 2009) and perennial woody species (Padilla and Encina, 2005; Yao et al., 2009; Orfanoudakis et al., 2010). In this study, LR numbers were significantly increased in the mycorrhizal seedlings, in line with our previous results (Chen et al., 2017), evidencing the important role of AMF in regulating LR formation. RNA-Seq represents a powerful tool to disentangle plant-AMF interaction at molecular level. With this technique, Shu et al. (2016) demonstrated that girdling regulated litchi mycorrhizal root development by altering carbohydrate metabolism (glyoxylate and dicarboxylate metabolism, citrate cycle, and pentose phosphate pathway). In grapevine roots, AMF significantly altered the expression of genes involved in nutrient transport, transcription factors, and cell wall-related genes (Balestrini et al., 2017). However, these work did not address how AMF regulated LR formation in woody plants. To gain insight into the regulatory network of LR formation induced by AMF, we performed RNA-Seq and analyzed differences in gene expression between the roots of non-mycorrhizal and mycorrhizal trifoliate orange seedlings. 909 DEGs were obtained, and according to Gene Ontology categories, 15 DEGs in the root tip were associated with cell wall reorganization (Supplementary Data 2), leading to continued root growth in mycorrhizal trifoliate orange (Opitz et al., 2016). Besides, DEGs related to oxidation-reduction process, transmembrane transport, kinase activity and membrane also possibly played roles in root development in response to AMF inoculation.

It is well accepted that AMF regulates LR formation via three pathways (see review by Fusconi, 2014), e.g., P metabolism, carbohydrate metabolism, and plant hormone metabolism. P is one of the most important constraints for plant growth and development in terrestrial ecosystems, especially in acid soils with low P availability (Xu et al., 2003; Kochian et al., 2004). In this study, shoot P content in the mycorrhizal plants was ~2 times that in the non-mycorrhizal plants, indicating the involvement of P metabolism in the regulation of LR formation by AMF. Actually, the 31 DEGs related to P metabolism demonstrate the active but complex P metabolism in roots following AMF inoculation. Phylogenetic analysis indicated that *LOC102613232* and *LOC102628168* encoded the mycorrhizal- inducible P transporter, positively responding to AMF colonization in trifoliate orange plants. In the same species, Shu et al. (2012) found that AMF increased the expression of *PtaPT4* and *PtaPT5* by up to 800-fold, indicating the homology of *LOC102613232* and *LOC102628168* with *PtaPT4* and *PtaPT*. In contrast, the down-regulated DEGs related to acid phosphatase are probably indicative of alleviated P starvation in mycorrhizal plants, because P deficiency induced the *PtPAP* expression and acid phsophatase activity in trifoliate orange plants (Shu et al., 2014). PLS-SEM analysis showed that P metabolism was the most influential pathway by which AMF regulated the LR formation in trifoliate orange.

AMF can enhance shoot photosynthesis in citrus via the activation of the light-harvesting complex family of proteins in photosystem II (Gao et al., 2016), and regulate the sucrose metabolism in shoots (Wu et al., 2015). AMF colonizing roots represent a strong sink for photosynthates, and drive the transport of photosynthates (mainly as sucrose) from shoots to roots. These shoot-derived carbohydrates fulfill a dual role, acting as metabolism substrates and as signaling molecules (Smeekens et al., 2010). In this study, the increased glucose and fructose contents in primary roots (Supplementary Figure 1B) by AMF probably contribute to metabolisms essential to LR formation. Moreover, 14 DEGs were enriched in “starch and sucrose metabolism” (Supplementary Figure 5), indicating the involvement of sugar metabolism in the regulation. However, we did not identify any DEG related to sugar signaling, which was proposed by Hammond and White (2011). Although Zhou et al. (2008) demonstrated that sugar signaling mediated the cluster root formation induced by P starvation in white lupin, it seems that sugar signaling was not involved in the LR formation in trifoliate orange. In contrast, DEGs related to polygalacturonase (PG), chitinase (CHI) and their analogs (PG-like protein, CHI-like protein) were drastically up-regulated in inoculated seedlings. It is reported that PG activity increased during the LR emergence, and played an important role in the LR outgrowth (Peretto et al., 1992). CHI catalyzes the hydrolysis of 1,4-β-linkages inside chitin, and thus degrade the fungal cell wall to limit pathogen invasion (Eckardt, 2008). In mycorrhizal root, class III chitinase genes were strongly induced (Krajinski and Frenzel, 2007), in accordance with our result that *LOC102607011* was up-regulated by AMF. According to Salzer et al. (2000), expression of class III chitinase genes were mycorrhiza-specific, while expression of defense-related chitinase genes (class I, II, and IV) were kept at a low and basal level in mycorrhiza. Recently, increasingly accumulated evidence indicate that CHI-like proteins may be also involved in the growth and developmental processes of plants (Kesari et al., 2015), including root morphogenesis (Hermans et al., 2010, 2011). It is noteworthy that sugar metabolism pathway negatively affect the LR formation as revealed by PLS-SEM analysis, probably indicating the competition for carbohydrate between AMF and LR formation. In overall, these data probably suggested that sugar metabolites were involved in the AMF regulated LR formation, but sugar signaling was not.

Plant hormones, especially auxin, play vital roles in LR development and RSA construction (Qu et al., 2016; Chen et al., 2017). In this study, among 25 DEGs related to plant hormones, 10 were associated with auxin (Figure 4C), demonstrating the primary role of auxin in LR formation regulated by AMF. It is evidenced that the biosynthesis of auxin in plant was promoted following AMF inoculation (Kaldorf and Ludwig-Müller, 2000; Alenazi et al., 2015), leading to increased LR formation. Another important plant hormone in this study was ethylene, involved in reducing LR formation (He et al., 2005; Singh et al., 2015), and seven DEGs related to ethylene were identified, four of which were ethylene-response transcription factors (ERF) (Figure 4C). In Arabidopsis, RNA interference-mediated suppression of *AtERF070* (*Arabidopsis thaliana Ethylene Response Factor070*), a Pi starvation induced TF belonging to the APETALA2/ETHYLENE RESPONSE FACTOR family, led to augmented LR development (Ramaiah et al., 2014). Three of 4 ERFs were down-regulated, consistent with the increasing LR number in inoculated plants. The positive effect of auxin and negative effect of ethylene were also confirmed in the PLS-SEM analysis. Despite the contribution of gibberellin (Gou et al., 2010), abscisic acid (Soucek et al., 2007; Hong et al., 2013), and cytokinin (Aloni et al., 2006; Laplaze et al., 2007) on LR development, there were few DEGs related to them in this study, especially the latter two plant hormones, indicating that they might be not involved in AMF's modification on LR formation.

Our data demonstrated that AMF increased LR formation in trifoliate orange. Interestingly, however, AMF only colonized large LR but did not colonize fine LR in rice (Fiorilli et al., 2015). With RNA-Seq technique, they found the down regulation of genes involved in AM establishment in fine LR compared with large LR, indicating some mechanisms suppressing AM symbiosis in fine LR. These findings highlight the complexity in the interaction between AMF and LR formation, and thus call for more intensive work in this aspect.

Recently, several studies demonstrated that in addition to sugars, lipids are another major C source delivered to AMF in mycorrhiza (Jiang et al., 2017; Luginbuehl et al., 2017). In this study, we identified 28 DEGs related to lipid biosynthesis and metabolism. Particularly, 4 DEGs related to fatty acid metabolism were up-regulated by AMF. Similarly, some DEGs related to fatty acid biosynthetic process and long-chain fatty acid metabolic process were up-regulated upon AMF colonization (Fiorilli et al., 2015). These indicated that AMF can alter fatty acid metabolism of the host plant by their requirement of lipids. Moreover, steroids act as early AM signals and play an important role in many aspects of AM symbiosis, e.g., hyphal penetration and arbuscule formation (Bucher, 2010). This may explain the up-regulation of 8 DEGs AMF in this study.

## Conclusion

To gain insights into the roles of P metabolism, sugar metabolism and plant hormones in the regulation of LR formation by AMF, we inoculated trifoliate orange seedlings with *Rhizophagus irregularis*. AMF inoculation strongly promoted the LR formation, as well as plant biomass and P concentration. RNA-Seq analysis revealed 909 DEGs in response to AMF inoculation, with 31, 32, and 23 DEGs related to P metabolism, sugar metabolism and plant hormones. These DEGs mainly encoded phosphate transporter, acid phosphatase, polygalacturonase, chitinase, auxin signaling, and ethylene signaling. PLS-SEM analysis indicates that AMF regulated LR formation mainly via the pathway of P metabolism in this study. In general, this transcriptomic data provide a global view of the pathways by which AMF regulates LR formation.

## Author contributions

WC, HZ, and QY designed the experiments. WC, JL, and PX performed the experiments. WC and PX carried out the bioinformatics analyses. WC, HZ, JC, and QY analyzed the data. WC and QY wrote the manuscript.

### Conflict of interest statement

The authors declare that the research was conducted in the absence of any commercial or financial relationships that could be construed as a potential conflict of interest.
