# Supplementary material for: Arbuscular Mycorrhizal Fungus Enhances Lateral Root Formation in Poncirus trifoliata (L.) as Revealed by RNA-Seq Analysis
Source: Front Plant Sci. 2017 Nov 29;8:2039. doi: 10.3389/fpls.2017.02039 (PMC5713035; doi:10.3389/fpls.2017.02039)

Supplementary Material

Title:

Arbuscular mycorrhizal fungus enhanced lateral root formation in *Poncirus trifoliata* (L.) as revealed by RNA-Seq analysis

Authors:

Weili Chen, Juan Li, Honghui Zhu, Pengyang Xu, Jiezhong Chen, Qing Yao*

* Correspondence: Qing Yao: yaoqscau@scau.edu.cn

Supplementary Tables and Figures

Supplementary Table 1 Primers of each gene in real-time quantitative RT-PCR

Gene ID	Gene Description	Sequence of primer（5'-3'）	Size of amplified product
LOC102607105	glucose-6-phosphate/phosphate translocator 2	F: ATTCAGCATTGGCAACACAA<br>R: CTTCACTGCTTTGCCTGTGA	147
LOC102608606	wall-associated receptor kinase-like 9	F: TACTCTTCTGGCTCCCCAAA<br>R: GACCCCTTTAGCATTGCTTG	150
LOC102610019	ABC transporter B family member 11-like	F: CAAACAAGCTCCTTGGCTTC<br>R: CACGCAGAAACTGGACTTCA	151
LOC102610425	auxin-responsive protein SAUR64	F: CAAAGGCCATTTTGTGGTCT<br>R: TGCTATCACAGGGCAGAGTG	155
LOC102610883	fructose-bisphosphate aldolase, cytoplasmic isozyme 1	F: GTCGAAATAGCCGGAACAAA<br>R: GACAATTCGGATGGCTCAGT	149
LOC102611582	protein NRT1/PTR FAMILY 4.5-like	F: TACACTCGTTGGGTTGCTTG	147

		R: CTTTTGGTCACGGCGTTTAT	
LOC102611714	ABC transporter G family member 17	F: AATTTGCAAGAACGGGATTG<br>R: ATATTCGTTCCTCCCCCAAC	154
LOC102615259	acid phosphatase 1	F: GGGGTGTCACTGCCATTATT<br>R: GATGAATCTGATGGGGGATG	148
LOC102624296	vacuolar cation/proton exchanger 3-like	F: GGGATGATAAGGGTCGTTCA<br>R: TCCAGAGTTCACCACAGCAG	150
LOC102624561	bidirectional sugar transporter SWEET4-like	F: CATTTTTGGAGCCCTCATGT<br>R: TCGAAACGAATGAGAGCGTA	150
LOC102626503	auxin response factor 24-like, transcript variant X2	F: AAATGCAATCAGGCTTGGAG<br>R: TCATAAGGTGCGAATGGTGA	151
LOC102630025	abscisic acid 8'-hydroxylase 3-like	F: ACCGTGAAATCCAAGCAAAC<br>R: TCAAAATCTTGAGCGGCTTT	151
LOC107177618	U-box domain-containing protein 11-like	F: CAGTTTCGGCACCCAGTAAT<br>R: GCGAGGTCTTCAGCTTCAGT	154

LOC107178343	transcriptional regulator TAC1-like	F: CAAGCTTTGGGAGGTCACAT<br>R: ATCGATGGCCTTATTGTGGA	149

Supplementary Table 2 DEGs related to phosphorus metabolism for RDA analysis and their correlations with LR number. Red and green fonts indicate the up-regulated and down-regulated DEGs, respectively. ‘*’, ‘**’ and ‘***’ mean significant differnce at *P*<0.05, *P*<0.01 and *P*<0.001, respectively.

Gene number	Gene ID	Annotation	Correlation efficiency with LR number			
			1st LR	2st LR	3rd LR	4st LR
1	LOC102624233	glycerol-3-phosphate 2-O-acyltransferase 6	0.392	**0.985****	**0.964****	**0.962****
2	LOC102613232	inorganic phosphate transporter 1-11	0.439	**0.991****	**0.973****	**0.944****
3	LOC102612787	glycerol-3-phosphate 2-O-acyltransferase 6-like	0.366	**0.980****	**0.958****	**0.970****
4	LOC102628168	probable inorganic phosphate transporter 1-3	0.445	**0.991****	**0.975****	**0.941****
5	LOC102624511	Serine/threonine-protein phosphatase PP1-like	0.483	**0.988****	**0.960****	**0.909***
6	LOC102619334	Serine/threonine protein phosphatase 2A	0.310	**0.963****	**0.940****	**0.984****
7	LOC102614267	acid phosphatase 1	0.735	**0.904***	**0.911***	0.674
8	LOC102623644	inositol-tetrakisphosphate 1-kinase 1-like	0.137	**0.847***	**0.857***	**0.952****
9	LOC107175387	heterodimeric geranylgeranyl pyrophosphate synthase large subunit 1	0.308	**0.898***	**0.835***	**0.886***
10	LOC102619887	protein phosphatase inhibitor 2-like	0.302	**0.952****	**0.939****	**0.970****
11	LOC102612161	probable protein phosphatase 2C 72	0.596	**0.935****	**0.955****	0.795

12	LOC102626737	sodium-dependent phosphate transporter 1-like	0.408	**0.84****	**0.977****	**0.957****
13	LOC102629248	glycerol-3-phosphate dehydrogenase [NAD(+)]	0.298	**0.955****	**0.942****	**0.977****
14	LOC102618747	Serine/threonine-protein phosphatase 6 regulatory ankyrin repeat subunit B-like	0.621	**0.975****	**0.987****	**0.848***
15	LOC102608061	inorganic phosphate transporter 2-1	0.050	**0.847***	**0.828***	**0.980***
16	LOC102620316	probable protein phosphatase 2C 40	0.555	**0.941****	**0.957****	**0.826***
17	LOC102618302	ectonucleotide pyrophosphatase/phosphodiesterase family member 3-like	0.476	**0.938****	**0.959****	**0.888***
18	LOC107176745	geranylgeranyl pyrophosphate synthase	0.355	**0.938****	**0.879***	**0.939****
19	LOC102610048	glycerol-3-phosphate dehydrogenase [NAD(+)] GPDHC1	0.429	0.683	0.764	0.576
20	LOC102611031	probable protein phosphatase 2C 24	0.414	**0.825***	**0.86***	0.800
21	LOC102617538	purple acid phosphatase 4	-0.392	**-0.896***	**-0.900***	**-0.839***
22	LOC102630359	probable purple acid phosphatase 20	-0.628	**-0.922****	**-0.971****	-0.785
23	LOC102620578	dCTP pyrophosphatase 1	-0.389	**-0.960****	**-0.949****	**-0.917***
24	LOC102618028	inositol-tetrakisphosphate 1-kinase 3	-0.329	**-0.910***	**-0.909***	**-0.878***
25	LOC102607430	purple acid phosphatase 2-like	-0.556	**-0.957****	**-0.982****	**-0.847***
26	LOC102620281	ent-copalyl diphosphate synthase	-0.660	**-0.920****	**-0.957****	-0.794
27	LOC102617018	inorganic pyrophosphatase 1	-0.572	**-0.968****	**-0.964****	**-0.859***
28	LOC102609811	inorganic phosphate transporter 1-4	-0.544	**-0.931****	**-0.963****	-0.813

29	LOC102625295	purple acid phosphatase 5	-0.604	**-0.901***	**-0.908***	**-0.813***
30	LOC102630609	probable glycerol-3-phosphate acyltransferase 8	-0.517	**-0.969****	**-0.963****	**-0.901***
31	LOC102615259	acid phosphatase 1	-0.220	**-0.828***	-0.780	**-0.829***

Supplementary Table 3 DEGs related to sugar metabolism for RDA analysis and their correlations with LR number. Red and green fonts indicate the up-regulated and down-regulated DEGs, respectively. ‘*’, ‘**’ and ‘***’ mean significant diffence at *P*<0.05, *P*<0.01 and *P*<0.001, respectively.

Gene number	Gene ID	Annotation	Correlation efficiency with LR number			
			1st LR	2st LR	3rd LR	4st LR
1	LOC102611569	polygalacturonase-like	0.682	**0.905***	**0.928****	0.715
2	LOC102621263	polygalacturonase-like	0.404	**0.915***	**0.934****	**0.882***
3	LOC107175540	pectinesterase-like	0.416	**0.983****	**0.972****	**0.949****
4	LOC102627325	hevamine-A	0.577	**0.987****	**0.976****	**0.863***
5	LOC102624297	acidic mammalian chitinase-like	0.338	**0.928****	**0.937****	**0.932****
6	LOC107174675	endochitinase EP3-like	0.468	**0.928****	**0.948****	**0.860***
7	LOC102624561	bidirectional sugar transporter SWEET4-like	0.772	0.768	0.802	0.497
8	LOC102626135	probable pectinesterase 53	0.229	**0.907***	**0.850***	**0.943****
9	LOC102607842	chitinase 4-like, transcript variant X1	0.412	**0.936****	**0.889***	**0.872***
10	LOC102607011	acidic endochitinase-like	0.627	**0.968****	**0.976****	**0.817***
11	LOC102624333	glucose-6-phosphate 1-dehydrogenase	0.484	**0.927****	**0.948****	**0.853***

12	LOC102621131	acidic mammalian chitinase-like	0.515	**0.963****	**0.973****	**0.875***
13	LOC102608623	endochitinase EP3	0.657	**0.930****	**0.965****	0.783
14	LOC102607007	alpha-glucosidase-like	0.246	**0.856***	**0.876***	**0.866***
15	LOC102612446	alpha-amylase	0.272	**0.949****	**0.908***	**0.971****
16	LOC102609716	bifunctional L-3-cyanoalanine synthase/cysteine synthase 1	0.366	**0.973****	**0.958****	**0.974****
17	LOC102610864	endochitinase EP3-like	0.470	**0.961****	**0.969****	**0.902***
18	LOC102619901	beta-hexosaminidase 2-like	0.441	0.793	**0.848***	0.693
19	LOC102626400	6-phosphogluconate dehydrogenase, decarboxylating 1	0.464	**0.961****	**0.970****	**0.897***
20	LOC102628532	malate dehydrogenas	0.370	**0.921****	**0.929****	**0.913***
21	LOC102616036	phosphoenolpyruvate carboxylase 4	0.462	**0.912***	**0.928****	**0.836***
22	LOC102630080	beta-fructofuranosidase, insoluble isoenzyme CWINV1	0.593	**0.917****	**0.950****	0.803
23	LOC102631481	probable fructokinase-6	0.220	**0.913***	**0.900***	**0.978****
24	LOC102625983	cysteine synthase-like	0.324	**0.870***	**0.880***	**0.880***
25	LOC102625409	glucose-1-phosphate adenylyltransferase small subunit 2	-0.558	**-0.857***	**-0.894***	-0.700
26	LOC102609095	pectinesterase/pectinesterase inhibitor PPE8B-like	-0.039	**-0.824***	-0.752	**-0.929****
27	LOC102624502	probable sucrose-phosphate synthase 4	-0.545	**-0.924****	**-0.967****	**-0.813***
28	LOC102629211	glucose-1-phosphate adenylyltransferase large subunit 1-like	-0.592	**-0.908***	**-0.939****	-0.751

29	LOC102616429	endoglucanase 2-like	-0.582	**-0.940****	**-0.981****	**-0.836***
30	LOC102607105	glucose-6-phosphate/phosphate translocator 2	-0.507	**-0.920****	**-0.945****	-0.806
31	LOC102610883	fructose-bisphosphate aldolase	-0.262	**-0.871***	**-0.854***	**-0.850***
32	LOC102578034	putative thermostable pectinesterase	-0.639	**-0.962****	**-0.981****	**-0.843***

Supplementary Table 4 DEGs related toplant hormone for RDA analysis and their correlations with LR number. Red and green fonts indicate the up-regulated and down-regulated DEGs, respectively. '*', '**' and '***' mean significant diffence at $P<0.05$, $P<0.01$ and $P<0.001$, respectively.

Gene number	Gene ID	Annotation	Correlation efficiency with LR number			
			1st LR	2st LR	3rd LR	4st LR
1	LOC102625856	gibberellin 2-beta-dioxygenase 8 transcript variant X2	0.757	**0.899***	**0.905***	0.661
2	LOC107178046	auxin-responsive protein SAUR64-like	0.634	**0.932****	**0.950****	0.773
3	LOC107178047	auxin-responsive protein SAUR68-like	0.652	**0.865***	**0.901***	0.688
4	LOC102610406	AP2-like ethylene-responsive transcription factor At1g16060	0.414	**0.988****	**0.969****	**0.954****
5	LOC102611011	AP2-like ethylene-responsive transcription factor AIL5 transcript variant X2	0.435	**0.990****	**0.962****	**0.943****
6	LOC102626503	auxin response factor 24-like transcript variant X2	0.271	**0.950****	**0.931****	**0.989****
7	LOC102610425	auxin-responsive protein SAUR64	0.434	**0.991****	**0.968****	**0.946****
8	LOC102630025	abscisic acid 8'-hydroxylase 3-like	0.425	**0.987****	**0.972****	**0.948****
9	LOC102616491	cytokinin dehydrogenase 3-like	0.546	**0.952****	**0.964****	**0.841***
10	LOC102622009	probable indole-3-acetic acid-amido synthetase GH3.1	0.370	**0.936****	**0.947****	**0.926****
11	LOC102614198	gibberellin 2-beta-dioxygenase	0.600	**0.981****	**0.990****	**0.867***

12	LOC102626389	AP2-like ethylene-responsive transcription factor At1g16060	0.443	**0.981****	**0.980****	**0.935****
13	LOC102625042	ethylene-responsive transcription factor ERF062	0.548	**0.867***	**0.899***	0.737
14	LOC102629869	auxin-induced protein 15A	0.458	**0.965****	**0.939****	**0.893***
15	LOC102614515	gibberellin 2-beta-dioxygenase 2	0.648	**0.933****	**0.943****	0.751
16	LOC102610790	indole-3-acetic acid-induced protein ARG2-like	0.536	**0.973****	**0.995****	**0.855***
17	LOC107177770	auxin-induced in root cultures protein 12	0.076	0.721	0.752	**0.838***
18	LOC102619305	auxin-responsive protein SAUR72	-0.470	**-0.966****	**-0.922****	**-0.896***
19	LOC102611406	abscisic acid receptor PYL4	-0.407	**-0.821***	**-0.833***	**-0.812***
20	LOC102623540	cytokinin riboside 5'-monophosphate phosphoribohydrolase LOG1-like	-0.307	**-0.941****	**-0.918****	**-0.939****
21	LOC102628717	gibberellin-regulated protein 1	-0.281	**-0.887***	**-0.903***	**-0.882***
22	LOC102619724	ethylene-responsive transcription factor ERF003	-0.352	**-0.920****	**-0.929****	**-0.882***
23	LOC102613600	auxin response factor 4 transcript variant X1	-0.551	**-0.948****	**-0.978****	**-0.835***
24	LOC102615809	ethylene-responsive transcription factor ERF023-like	-0.418	**-0.926****	**-0.888***	**-0.870***
25	LOC102622538	ethylene-responsive transcription factor ERF014	-0.357	-0.771	-0.698	-0.700

Supplementary Table 5 DEGs related to lipid biosynthesis and metabolism. 'Fold Change' indicates the ratio of expressions in T and C treatment of the same gene. Red and green fonts indicate the up-regulated and down-regulated DEGs, respectively.

Gene ID	FoldChange	Annotation	KEGG Pathway
LOC102630609	0.239	probable glycerol-3-phosphate acyltransferase 8	GlyceroLipid metabolism
LOC102625429	0.304	linoleate 13S-lipoxygenase 2-1 chloroplastic	Linoleic acid metabolism
LOC102606741	0.325	phospholipase D zeta 1 transcript variant X3	GlycerophosphoLipid metabolism
LOC102614331	0.338	monogalactosyldiacylglycerol synthase 2 chloroplastic	GlyceroLipid metabolism
LOC102629931	0.347	linoleate 13S-lipoxygenase 2-1 chloroplastic-like	Linoleic acid metabolism
LOC102625710	0.374	linoleate 13S-lipoxygenase 2-1 chloroplastic	Linoleic acid metabolism
LOC102608065	0.384	putative phospholipid:diacylglycerol acyltransferase 2 transcript variant X2	GlyceroLipid metabolism
LOC102625340	0.431	stearoyl-[acyl-carrier-protein] 9-desaturase 5 chloroplastic-like	Fatty acid metabolism
LOC102627239	0.443	delta(12)-fatty-acid desaturase FAD2 transcript variant X1	Fatty acid metabolism
LOC102626031	0.449	stearoyl-[acyl-carrier-protein] 9-desaturase 5 chloroplastic-like	Fatty acid metabolism
LOC102619040	0.484	phospholipase D alpha 4	GlycerophosphoLipid metabolism
LOC102608487	2.164	non-specific phospholipase C2 transcript variant X2	Inositol phosphate metabolism
LOC102610048	2.217	glycerol-3-phosphate dehydrogenase [NAD(+)] GPDHC1 cytosolic	GlycerophosphoLipid metabolism
LOC102625150	3.600	cycloartenol synthase-like transcript variant X1	Steroid biosynthesis

LOC102628234	4.958	cytochrome P450 71A1-like	Arachidonic acid metabolism
LOC102629248	6.865	glycerol-3-phosphate dehydrogenase [NAD(+)]	GlycerophosphoLipid metabolism
LOC102611163	6.940	delta(12)-fatty-acid desaturase FAD2 transcript variant X2	Fatty acid metabolism
LOC102626302	14.315	3-oxoacyl-[acyl-carrier-protein] synthase I chloroplastic-like	Fatty acid metabolism
LOC102618665	16.371	palmitoyl-acyl carrier protein thioesterase chloroplastic-like transcript variant X1	Fatty acid metabolism
LOC102624111	80.025	squalene epoxidase 3-like	Steroid biosynthesis
LOC102614438	152.465	cycloartenol synthase 2-like	Steroid biosynthesis
LOC102629087	790.536	neutral ceramidase-like	SphingoLipid metabolism
LOC102607692	1106.717	triacylglycerol lipase 2-like	Steroid biosynthesis
LOC102608280	1617.213	triacylglycerol lipase 2-like	Steroid biosynthesis
LOC102607995	inf	triacylglycerol lipase 2-like	Steroid biosynthesis
LOC102614721	inf	squalene epoxidase 3-like	Steroid biosynthesis
LOC102615022	inf	squalene epoxidase 3-like	Steroid biosynthesis
LOC102612001	inf	3-oxoacyl-[acyl-carrier-protein] synthase II chloroplastic-like	Fatty acid metabolism

Supplementary Table 6 Total effects and Bootstrapping analysis in PLS-SEM. T statistics higher than 1.96 were significant at 5% (Hair et al., 2011).

Pathways	Original Sample (Total effects)	Sample Mean	Standard Deviation	Standard Error	T Statistics
AMF→Auxin	0.990	0.990	0.000	0.000	11649.350
AMF→Ethylene	0.987	0.987	0.000	0.000	12795.970
AMF→Lateral root	0.969	0.969	0.000	0.000	4247.607
AMF→Phosporus	0.991	0.991	0.000	0.000	15770.800
AMF→Sugar	0.995	0.995	0.000	0.000	75812.320
Auxin→Lateral root	0.746	0.748	0.050	0.050	14.987
Ethylene→Lateral root	-0.383	-0.385	0.005	0.005	72.787
P→Lateral root	1.854	1.854	0.034	0.034	54.410
sugar→Lateral root	-1.084	-1.087	0.023	0.023	46.474

Supplementary Table 7 PLS-SEM results quality criteria. All latent variables were significant and goodness-of-fit measures such as average variance extracted (AVE; Indicator for converge validity) and composite reliability (indicator for internal consistency reliability) were equal or higher than 0.5 and 0.7 (Hair et al., 2011).

	AVE	Composite Reliability	R Square
AMF	0.999	1.000	Empty cell
Auxin	0.871	0.982	0.981
Ethylene	0.901	0.985	0.973
Lateral root	0.810	0.971	1.000
Phosphorus	0.887	0.996	0.981
Sugar	0.886	0.996	0.991

Supplementary Figure 1 Lateral root numbers and sugar contents in trifoliate orange roots. (A) Lateral root numbers; (B) Sugar contents in primary root (PR); (C) Sugar contents in lateral root (LR). C: non-mycorrhizal treatment, T: mycorrhizal treatment; '*' and 'ns' mean significant difference at P<0.05 and no significant difference, respectively.

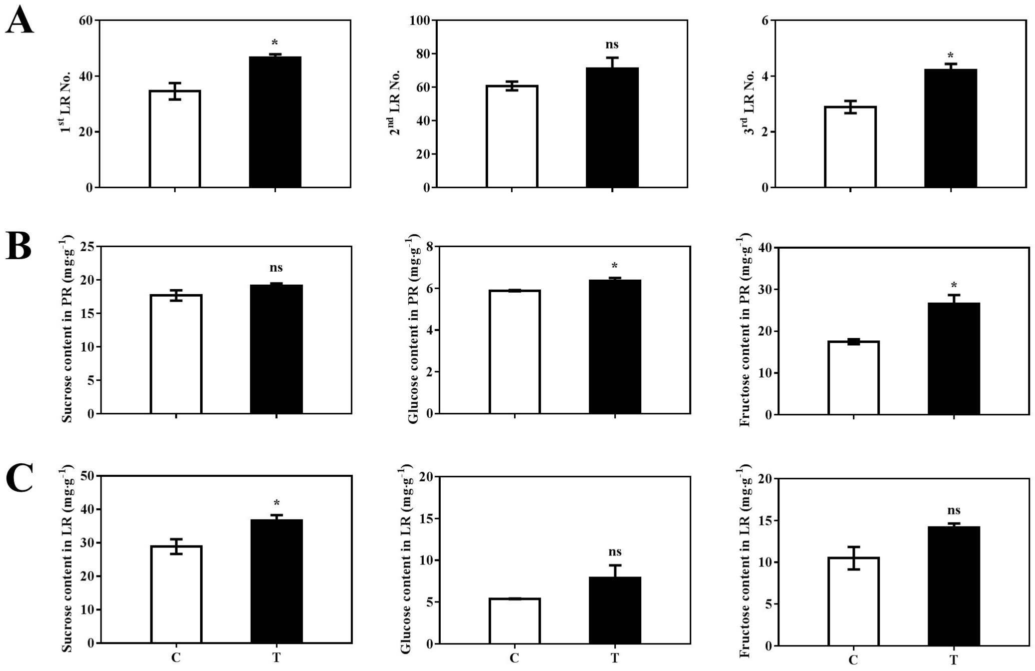

Supplementary Figure 2 Validation of RNA-seq results by qRT-PCR. Data from qRT-PCR of 14 selected genes were means of three replicates and bars represent Standard Error. RPKMs from RNA-seq were means of three replicates. C: non-mycorrhizal treatment, T: mycorrhizal treatment.

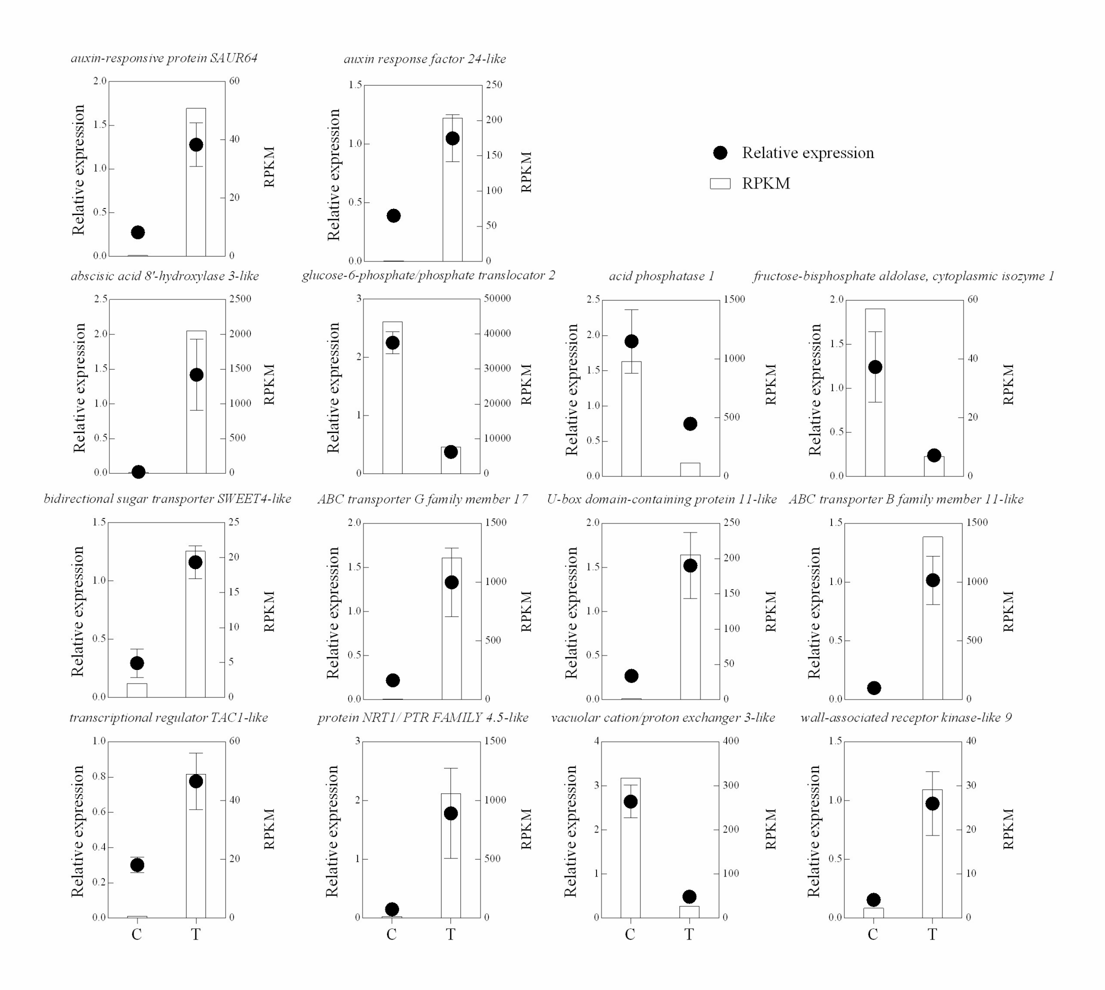

Supplementary Figure 3 Coefficient analysis of fold change data between qRT-PCR and RNA-seq. Scatterplots were generated by the log2(expression ratios) from RNA-seq (x-axis) and qRT-PCR (y-axis).

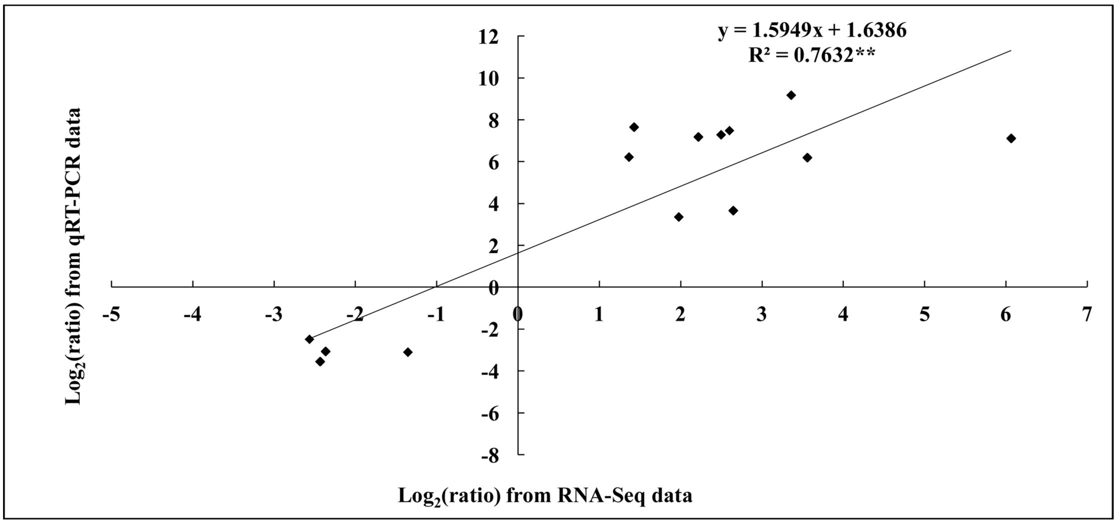

Supplementary Figure 4 Functional categories of differentially expressed genes according to GO terms.

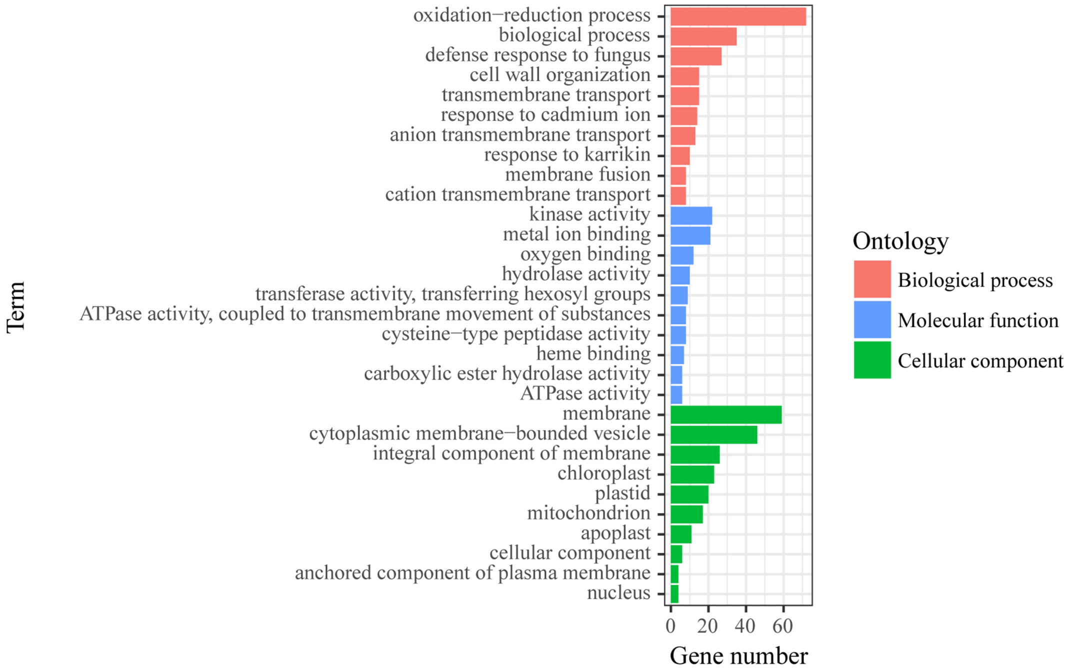

Supplementary Figure 5 The partial results of KEGG pathway analysis.

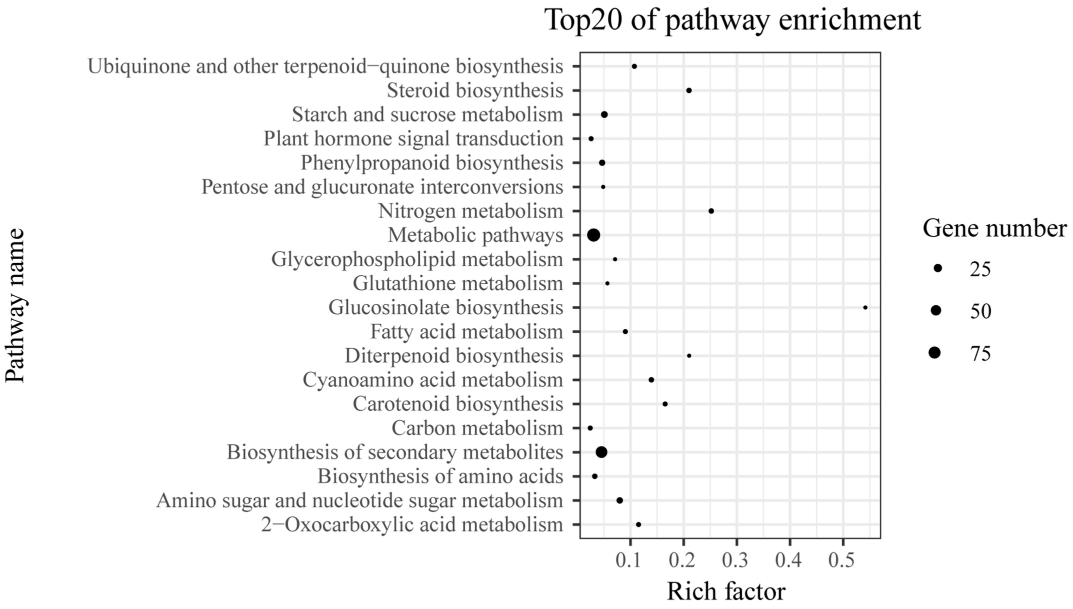

Supplementary Figure 6 Phylogenetic relationship of *PT* genes in DEGs of trifoliate orange roots in this study with *PT* genes (*PtaPT4*, *PtaPT5*) induced by AMF according to Shu et al. (2012).

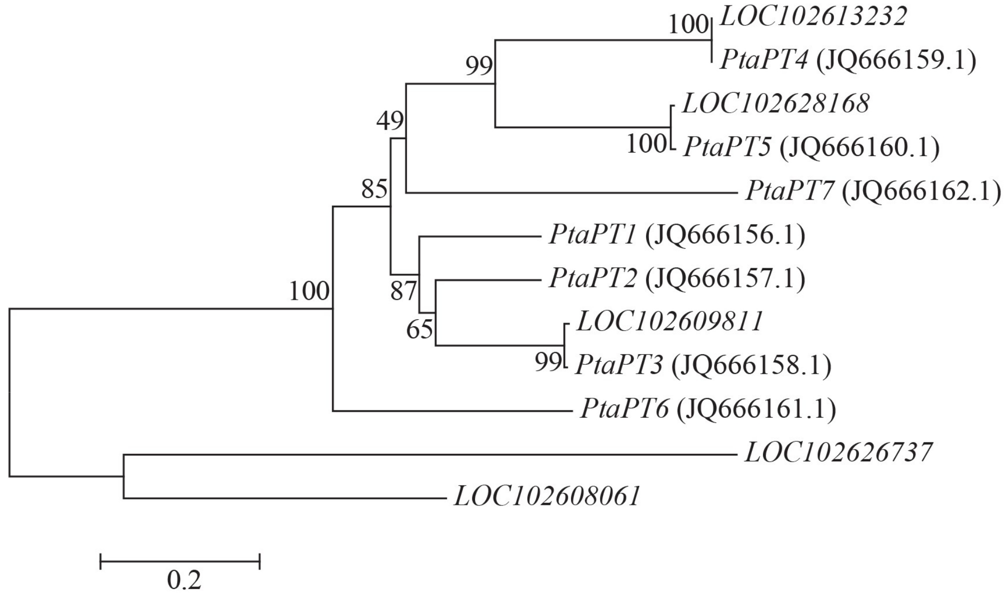

Supplement: Supplementary file 1 [file Presentation1.PDF]
